# Reply to De Luca et al. Comment on “Marra et al. Metastatic Renal Cell Carcinoma to the Soft Tissue 27 Years after Radical Nephrectomy: A Case Report. *Medicina* 2023, *59*, 150”

**DOI:** 10.3390/medicina59050835

**Published:** 2023-04-25

**Authors:** Carmine Alfano, Caterina Marra, Paola Pentangelo, Alessandra Ceccaroni, Donato Troisi, Luigi Losco

**Affiliations:** 1 Plastic Surgery Unit, Department of Medicine, Surgery and Dentistry, University of Salerno, Baronissi, 84081 Salerno, Italy; 2 ORL Unit, Azienda Ospedaliera Universitaria OO.RR. San Giovanni di Dio e Ruggi d’Aragona, Via S. Leonardo 1, 84131 Salerno, Italy

We appreciated the comments of De Luca and colleagues [1] regarding our recently published article [2]. We would like to clarify two points. Firstly, the metastasis described in our case report was localized in the subcutaneous tissue, it was not a bone metastasis. In fact, the ultrasound and the MRI documented that the mass was leaning against the bony profile, without infiltrating it. Secondly, as an elucidation of our report, we have to detail that the development of most cutaneous metastases takes place within five years from the initial diagnosis and after nephrectomy [3,4,5].

We agree with the authors regarding the surgical approach of pulsating intraoral metastases from renal cell carcinoma (RCC); preoperative vascular embolization should be performed in order to prevent massive hemorrhage during biopsy and surgery. However, in our case, the mass was not pulsatile and only a modest contrast enhancement was reported on imaging exams; indeed, a desmoid-type benign lesion was suspected.

Cutaneous and subcutaneous RCC metastases, as reported by Al Abdrabalnabi et al. [6], can mimic common dermatological disorders, such as lipoma, thus making identification difficult. This type of misdiagnosis could be avoided by submitting the patient to a longer follow-up time with a multidisciplinary team; unfortunately, as detailed in our report, the patient discontinued a proper oncologic follow-up due to the COVID-19 pandemic outbreak.

Metastasectomy in patients with RCC has never been analyzed in clinical randomized studies. [7] However, several recent retrospective studies have been performed to evaluate the effectiveness of metastasectomy in the context of improved systemic therapy with remarkable results in terms of cancer-specific survival and overall survival [8,9].

The relation between targeting agents and metastasectomy is not defined at present. However, in selected patients with a limited metastatic burden after systemic therapy, metastasectomy seems feasible with an acceptable morbidity [10]. In a multi-institutional review, Karam et al. [11] identified 22 patients treated with metastasectomy after targeted therapy. At a median follow-up of 109 weeks, 21 patients were alive and 1 died of renal cell carcinoma 105 weeks after metastasectomy. In our case, the Tumor Board opted to start targeted therapy treatment and consider metastasectomy after the mass had shrunk, leading to an easier complete removal.

Most of all, we thank the authors for sharing their therapeutic approach to solitary, accessible RCC metastatic lesion in head and neck and look forward to learning more about their relevant results; moreover, we agree that patients with a history of RCC should undergo proper monitoring even after a long disease-free survival.
